# Hsa_circ_0006988 Promotes Sorafenib Resistance of Hepatocellular Carcinoma by Modulating IGF1 Using miR-15a-5p

**DOI:** 10.1155/2022/1206134

**Published:** 2022-12-24

**Authors:** Rui Qiu, Zhifeng Zeng

**Affiliations:** Second General Surgery, Xinyu People's Hospital, Xinyu, China

## Abstract

**Background:**

Hepatocellular carcinoma (HCC) is the most frequently occurring cancer and contributes to the largest number of cancer-associated deaths worldwide. Recent evidence suggests that circular RNAs (circRNAs), which are critical for HCC etiology and metastasis, are distinctly modulated in HCC. Nevertheless, the underlying mechanism of circRNA-mediated sorafenib resistance (SOR) in HCC is yet to be determined.

**Methods:**

The hsa_circ_0006988, IGF1, and miR-15a-5p contents were quantified via ELISA and quantitative real-time polymerase chain reaction (qRT-PCR), respectively. Cell Counting Kit-8 (CCK-8) was used for the IC50 evaluation. Lastly, associations among hsa_circ_0006988, IGF1, and miR-15a-5p were validated through dual-luciferase reporter (DLR) and RNA immunoprecipitation (RIP) assays.

**Results:**

Herein, a new circRNA, hsa_circ_0006988, was identified, and its levels were markedly enhanced in SOR-resistant (SOR-R) HCC tissues. Functionally, hsa_circ_0006988 strongly suppressed SOR toxicity *in vitro*. Our examination of the signaling pathway revealed that hsa_circ_0006988 sequestered miR-15a-5p, a negative modulator of IGF1, thus suggesting that hsa_circ_0006988 deficiency diminished SOR resistance of HCC, and this action utilized the release of excess miR-15a-5p, which suppressed IGF1 levels. Moreover, miR-15a-5p overexpression reversed the hsa_circ_0006988-mediated SOR-R and enhanced IGF1 levels in HCC cells.

**Conclusion:**

Hsa_circ_0006988 partly promoted the SOR-R of HCC cells through miR-15a-5p sequestering and upregulation of IGF1 levels.

## 1. Introduction

With an 18% 5-year overall survival (OS) rate and a 65–80% postsurgical resurgence, hepatocellular carcinoma is the most severe form of primary liver cancer [1]. Recently, new therapeutic strategies such as cancer immunosuppressive therapy have improved patient survival, and the combination of an immune checkpoint inhibitor (ICI) and VEGF inhibitor is targeted as the first-line treatment for advanced HCC [2–4]. In terms of treatment, the multikinase inhibitor sorafenib (SOR) displays superior performance in alleviating HCC. However, HCC patients still suffer poor outcomes due to acquired resistance [5, 6]. SOR is an essential factor that limits the long-term OS of HCC patients. Hence, there is an urgent need for new therapeutic candidates to minimize sorafenib resistance (SOR-R) in HCC.

As covalently closed loop structures (CCLS) developed via back-splicing, circular RNAs (circRNAs) were found to be deficient in both a polyadenylated tail and 5′–3′ polarity [7, 8]. Emerging evidence revealed multiple physiological and pathophysiological activities modulated by circRNAs, such as alternative splicing [9], sequestering microRNA (miRNA) [7], and managing protein-RNA associations and gene expressions [10]. Their closed configuration offers circRNAs enhanced stability and advantages in resistance to RNA destruction. Recent post-transcriptional modulation has reported circRNAs associated with miRNAs as competing endogenous RNAs (ceRNAs) [11–13]. Similarly, circRNA plays an important role in angiogenesis and immune escape. For example, gastric cancer-derived exosomes mediate the delivery of circRNA to promote angiogenesis by targeting the miR-29a/VEGF axis in endothelial cells [14]. Epstein-Barrvirus-encoded circular RNA CircBART2.2 promotes the immune escape of nasopharyngeal carcinoma by regulating PD-L1 [15]. Moreover, aberrant circRNA levels were confirmed in various malignancies, namely HCC. Furthermore, in HCC, certain reports also identified multiple circRNAs, like circRHOT1 [16] and cSMARCA5 [17]. However, their significance and signaling pathways in SOR-R in HCC remain unidentified.

Herein, we examined the differentially regulated circRNAs in SOR-R HCC tissues using GEO circRNA arrays. Our screening uncovered hsa_circ_0006988 as preserved and markedly enhanced circRNA in SOR-R HCC cell lines and tissues. Loss- and gain-of-function assessments revealed that hsa_circ_0006988 induced SOR-R in HCC tissues. We demonstrated that hsa_circ_0006988 suppression sensitized HCC cells to SOR by modulating miR-15a-5p and its downstream IGF1 levels. Our conclusions will enable a novel understanding of the hsa_circ_0006988-induced mechanisms in tumorigenesis and SOR-R in HCC.

## 2. Materials and Methods

### 2.1. Patients and Tissue Samples

Matched fresh HCC tissues and corresponding ANTs were obtained from 156 HCC patients and were maintained in liquid nitrogen until further analysis. The patients were separated into sorafenib-sensitive (SOR-S, *n* = 82) and SOR-R cohorts (*n* = 74), following 2 regimens of SOR supplemental therapy. Tumor samples were acquired via surgery before the initiation of therapeutic intervention. This work received ethical approval from Xinyu People's Hospital, whereas a written informed consent form was obtained from all participants before the operation.

### 2.2. Cell Culture and Reagents

The Chinese Academy of Sciences Cell Bank Type Culture Collection was contacted for HCC, HepG2, and Huh-7 cancer cell lines and the normal human liver cell line LO2. The cells were maintained in DMEM and RPMI-1640 culture media (Gibco, Carlsbad, CA, USA) supplemented with 10% fetal bovine serum (Gibco) at 37°C and in a 5% CO_2_ incubator SOR (BAY 43-9006) provided by MedChem Express. It was resuspended in DMSO at <0.1%. To establish SOR-R hepatoma cells, HepG2 and Huh-7 cells were maintained in 1 mmol/L of SOR and gradually elevated by 0.5 mmol/L per month (up to 5 mmol/L) over a period of 10 months. Subsequently, two SOR-R cell lines were established, which were subsequently termed SOR-R HepG2 (SR-HepG2) and SOR-R Huh7 (SR-Huh7).

### 2.3. Cell Incorporation

The miR-15a-5p mimic (miR-M), hsa_circ_0006988, si-IGF1, and inhibitor (miR-I) siRNAs were prepared by RiboBio (Guangzhou, China). siRNA (50 nM), miR-M, and miR-I (50 nM) incorporations were performed using Lipofectamine 3000 (Invitrogen, Carlsbad, USA), following kit directions.

## 3. RT-qPCR

SYBR green kit (Takara, Dalian, China) was used to evaluate cDNA, and qRT-PCR was conducted via equipment obtained from Bio-Rad Laboratories (Berkeley, USA). The relative gene expression was computed using the 2^−ΔΔCt^ method. The hsa_circ_0006988 and IGF1 expressions were adjusted to GAPDH, and the miR-15a-5p expression was adjusted using U6 levels.

### 3.1. MTT Assay

After siRNA transfection, 5000 SR-HepG2 and SR-Huh7 cells were seeded in a 96-well plate and treated with SOR for 48 hours. Next, 2 mg/mL of MTT reagent (Sigma-Aldrich) was added to the wells for an additional 4 h. Lastly, all formazan formations were resuspended in 100 *μ*l of dimethylsulfoxide prior to the optical density (OD) assessment at 570 nm on a microplate reader. The SOR IC50 was determined using GraphPad Prism 7 (GraphPad Software, San Diego, USA).

### 3.2. Dual-Luciferase Reporter (DLR) Assay

The hsa_circ_0006988 or IGF1 fragments with miR-15a-5p docking sites were cloned into psiCHECK2 (Promega, Fitchburg, WI) to establish WT-hsa_circ_0006988 and WT-IGF1 luciferase reporter plasmids, respectively. We next synthesized the corresponding luciferase reporter vectors (MUT-IGF1 and MUT-hsa_circ_0006988). Following this, the specified vector and miRNA NC or miR-M were cotransferred into 293T cells. After 48 h, the DLR Assay Kit (Promega) was used to assess the activity.

### 3.3. RNA Pull-Down Assay

Biotinylated miR-15a-5p (bio-miR-15a-5p) or bio-NC was introduced into SR-HepG2 and SR-Huh7 cells. Following a 48 h incubation and cell lysis, the lysate was incubated with streptavidin-coupled magnetic beads (Invitrogen) for 2 h. Subsequently, following RIP of the biotin-associated RNA, the hsa_circ_0006988-interacting RNAs were examined via RT-qPCR following the extraction of associated RNAs.

### 3.4. RNA Immunoprecipitation (RIP) Assay

EZ-Magna RIP™ RNA-Interacting Protein Immunoprecipitation Kit (Millipore, Billerica, MA, USA) was utilized for the RIP assay. 293T cells underwent lysis in RIP lysis buffer with RNase inhibitor (Millipore). A 100 *μ*L cell lysate was treated with RIP with magnetic beads coated antibody. The hsa_circ_0006988 and miR-15a-5p, which then precipitated prior to evaluation via RT-qPCR.

### 3.5. Elisa Assay

The IGF1 expression was predicted in various culture media using an ELISA kit (R&D Systems) following kit directions. The presented results are the mean of 3 distinct experiments.

### 3.6. Statistical Analysis

Data are provided as the mean + standard deviation of 3 replicates, assessed via Student's *t*-test and a one-way analysis of variance (ANOVA). The Spearman rank correlation was employed to assess the associations among hsa_circ_0006988, IGF1, and miR-15a-5p transcript contents in HCC samples. *P* < 0.05 was considered the significance cut-off.

## 4. Results

### 4.1. Hsa_circ_0006988 Levels Were Correlated with SOR-Rof HCC

**Figure 1:**
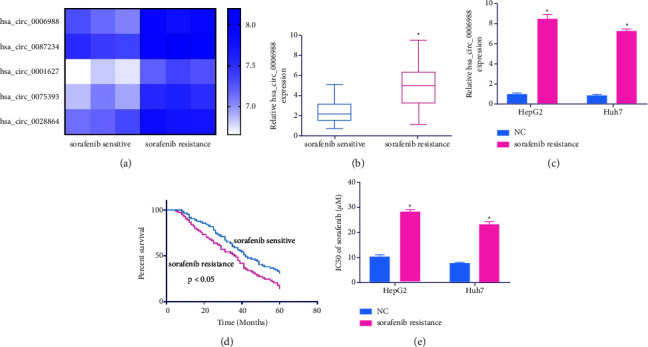
Hsa_circ_0006988 expression is associated with sorafenib resistance (SOR-R) in HCC. (a) Hierarchical clustering analyses were used to detect differences in circRNA expression profiles between SOR-R and sorafenib-sensitive (SOR-S) cells. (b) Hsa_circ_0006988 levels, via RT-qPCR, in sensitive and resistant tissues. (c) Hsa_circ_0006988 levels, via RT-qPCR, in HepG2, Huh7, SR-HepG2, and SR-Huh7 cells. (d) Association between hsa_circ_0006988 and overall survival (OS) of HCC patients. (e) The IC50s of SOR. The presented data are the mean of 3 replicates, and ^*∗*^*p* < 0.05.

Our analysis of circRNA expressions in 3 SOR-R and 3 SOR-S samples from the GSE101850 GEO microarray data identified 250 highly expressed circRNAs in HCC patients, among which the top 5 are presented in Figure 1(a). The hsa_circ_0006988 levels were markedly enhanced in SOR-R cells and tissues relative to SOR-S cells and tissues (Figures 1(b) and 1(c)). Moreover, the 5-year OS rate of the SOR-R patients with augmented expression was markedly reduced relative to the SOR-S (Figure 1(d)). We also demonstrated that the SOR of IC50 was enhanced in SOR-R cells relative to normal cells (Figure 1(e)).

### 4.2. Hsa_circ_0006988 Silencing Suppressed SOR-R in HCC Cells

**Figure 2:**
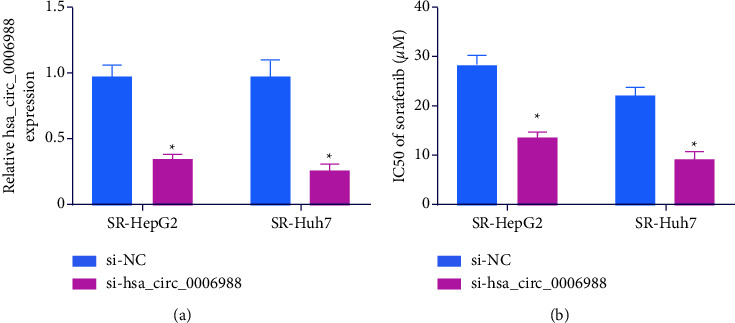
Hsa_circ_0006988 silencing suppresses sorafenib resistance (SOR-R) in SOR-R HCC cells. (a) Hsa_circ_0006988 quantification, via RT-qPCR, following silencing. (b) IC50s of SOR. The presented data are mean of 3 replicates, and ^*∗*^*p* < 0.05.

To elucidate the hsa_circ_0006988-mediated regulation of SOR-R in HCC cells, we incorporated SR-Huh7 and SR-HepG2 cells with si-hsa_circ_0006988. Our RT-qPCR data revealed that hsa_circ_0006988 levels exhibited a marked decrease in si-hsa_circ_0006988-incorporated SR-Huh7 and SR-HepG2 cells in comparison with controls (Figure 2(a)). Based on the MTT assay, the IC50 of SOR was drastically reduced in si-hsa_circ_0006988 incorporated SR-HepG2 and SR-Huh7 cells (Figure 2(b)). This evidence suggested a strong influence of hsa_circ_0006988 silencing in the SOR-R of HCC.

### 4.3. Hsa_circ_0006988 May be Employed to Sequester miR-15a-5p

**Figure 3:**
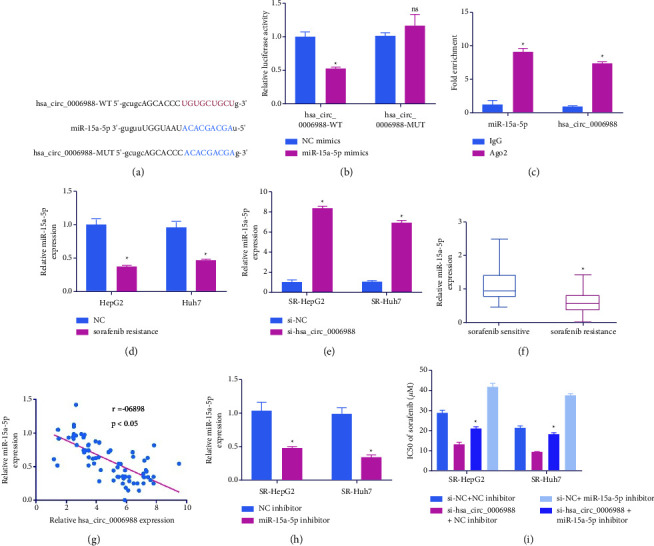
Hsa_circ_0006988 serves as a miR-15a-5p sponge. (a) Predicted docking sites for hsa_circ_0006988 and miR-15a-5p. (b) Direct binding between hsa_circ_0006988 and miR-15a-5p, as evidenced by the DLR assay. (c) RIP data confirming the hsa_circ_0006988 interaction with miR-15a-5p. (d) MiR-15a-5p levels, via RT-qPCR, in HepG2, Huh7, SR-HepG2, and SR-Huh7 cells. (e) MiR-15a-5p levels, via RT-qPCR, in sorafenib-resistant (SOR-R) cells incorporated with si-NC or si-hsa_circ_0006988. (f) MiR-15a-5p levels, via RT-qPCR, in sensitive and resistant tissues. (g) Association between hsa_circ_0006988 and miR-15a-5p, as evidenced by Spearman's correlation coefficient. (h) MiR-15a-5p levels, via RT-qPCR, in SOR-R cells incorporated with NC, or miR-15a-5p inhibitor (miR-I). (i) IC50s of SOR. The presented data are the mean of 3 replicates, and ^*∗*^*p* < 0.05.

To estimate the potential miR docking sites within hsa_circ_0006988 (Figure 3(a)), we conducted a DLR assay. The data demonstrated that miR-Ms strongly reduced hsa_circ_0006988-WT DLR activity (Figure 3(b)). Moreover, RIP analysis suggests that hsa_circ_0006988 and miR-15a-5p exhibit overt enrichment in the Ago2 cells relative to the lgG cells (Figure 3(c)). Furthermore, the miR-15a-5p expressions in SR-Huh7 and SR-HepG2 cells were strongly diminished relative to HepG2 and Huh7 cells (Figure 3(d)). Hsa_circ_0006988 deficiency enhanced miR-15a-5p expression (Figure 3(e)). Relative to SOR-S, the miR-15a-5p contents were significantly diminished in SOR-R tissues (Figure 3(f)). The miR-15a-5p content was inversely related to the hsa_circ_0006988 levels (Figure 3(g)). Following miR-I incorporation into cells, miR-15a-5p was strongly suppressed (Figure 3(h)). Moreover, hsa_circ_0006988 deficiency inhibited the IC50 of SOR, and IC50 reduction was restored with miR-I incorporation into SR-HepG2 and SR-Huh7 cells (Figure 3(i)).

### 4.4. MiR-15a-5p Targeting IGF1 to Inhibit Chemoresistance in SOR-RHCC Cells

**Figure 4:**
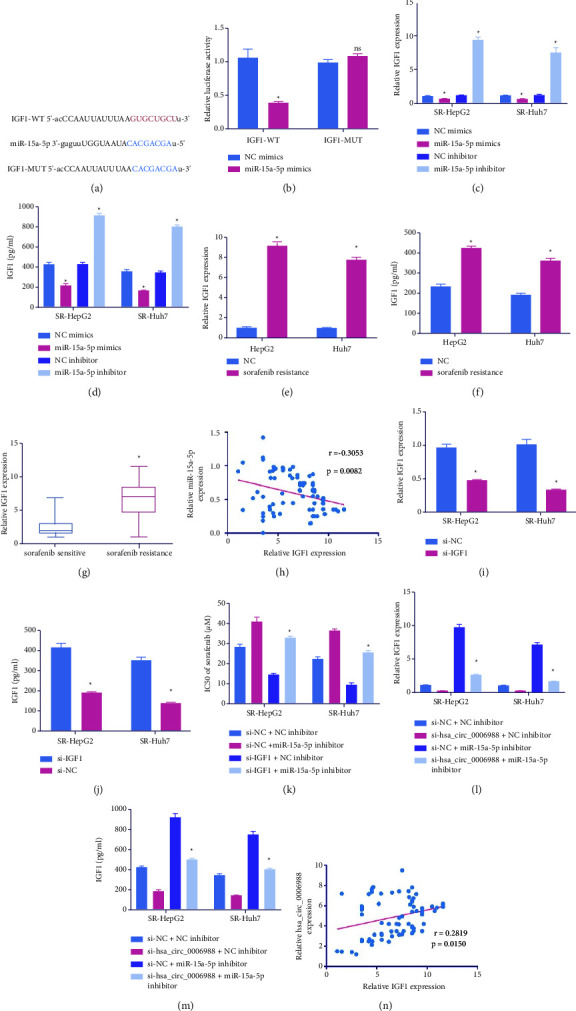
MiR-15a-5p targets IGF1 to inhibit chemoresistance in sorafenib-resistant (SOR-R) HCC cells. (a) Predicted docking sites for IGF1 and miR-15a-5p. (b) IGF1 and miR-15a-5p interaction validation, via DLR assay. IGF1 levels, via RT-qPCR (c) and ELISA assay (d). IGF1 levels via RT-qPCR (e) and ELISA assay (f), in HepG2, Huh7, SR-HepG2, and SR-Huh7 cells. (g) IGF1 levels, via RT-qPCR, in sensitive and resistant tissues. (h) IGF1 and miR-15a-5p association, via Spearman's correlation coefficient. IGF1 levels, via RT-qPCR (i) and ELISA assay (j). (k) IC50s of SOR. IGF1 levels, via RT-qPCR (l) and ELISA assay (m). (n) Hsa_circ_0006988 and IGF1 association, via Spearman's correlation coefficient. The presented data are mean of 3 replicates, and ^*∗*^*p* < 0.05.

We next estimated candidate miR-15a-5p target genes using an online estimation tool in an attempt to discern the underlying mechanism whilst identifying the docking sites of IGF1 on miR-15a-5p (Figure 4(a)). Employing DLR assay, we demonstrated that miR-Ms vastly reduced IGF1-WT fluorescence activity (Figure 4(b)). The IGF1 levels were diminished following miR-Ms incorporation and were elevated following miR-I incorporation (Figures 4(c) and 4(d)). Additionally, the IGF1 levels in SR-Huh7 and SR-HepG2 cells were elevated relative to the HepG2 and Huh7 cells (Figures 4(e) and 4(f)). Also, compared to SOR-S, the IGF1 expression was strongly enhanced in SOR-R tissues (Figure 4(g)). The IGF1 expression was negatively associated with the hsa_circ_0006988 levels (Figure 4(h)). Following si-IGF1 introduction into cells, IGF1 expression was strongly diminished (Figures 4(i) and 4(j)). Our findings revealed that the miR-15a-5p deficiency abrogated the IC50 of SOR, and the IC50 reduction was restored by the si-IGF1 incorporation into SR-Huh7 and SR-HepG2 cells (Figure 4(k)). Additionally, miR-15a-5p suppression reversed the hsa_circ_0006988- depletion and its effect on IGF1 expression (Figures 4(l) and 4(m)). The hsa_circ_0006988 levels were directly associated with IGF1 transcript levels (Figure 4(n)).

## 5. Discussion

Herein, we evaluated the significance of circRNAhsa_circ_0006988 on the SOR chemosensitivity of HCC and demonstrated the modulatory signaling behind the miR-15a-5p/IGF1 axis. Our analysis shows that elevated hsa_circ_0006988 levels enhanced the SOR-R of hepatocellular carcinoma cells. Hsa_circ_0006988 serves as a molecular sequester of miR-15a-5p, which, in turn, disrupts the suppressive effect of miRNA on IGF1. In addition, using DLR and RIP assays, we demonstrated a strong association among hsa_circ_0006988, miR-15a-5p, and IGF1. Collectively, this evidence indicated that hsa_circ_0006988 modulates the SOR-R of HCC, which, in turn, promotes HCC progression.

Over the past decade, drug interventions have markedly enhanced the OS of hepatocellular carcinoma cells in patients with complex diseases. An oral multikinase inhibitor, SOR, is a proliferation and angiogenesis suppressor, and it does so by modulating Raf-1, BRAF Flt3, PDGFR-b, and VEGFR-2/3 [18, 19]. SOR, an FDA-approvedanti-HCC therapeutic agent, is highly efficacious against HCC [20]. SOR and NK cells might improve the outcome of applied therapeutic approaches for HCC patients [21]. However, a majority of patients progress to develop drug-resistant diseases, thereby resulting in poor patient outcomes. The underlying mechanism behind the SOR-R of HCC is rather complicated. To date, the signaling pathways associated with HCC drug resistance remain undiscovered.

There has been much focus on circRNA, miRNA, and long noncoding RNA (lncRNA) in recent years [8]. The circRNA has a CCLS and does not encode proteins [22]. Emerging evidence suggests that circRNA modulates numerous physiological and pathological activities, such as proliferation, cellular differentiation, angiogenesis, metabolic stress responses, and cell death [23]. Impaired circRNAs behave like tumor suppressor oncogenes in their modulation of cancer development and progression, including HCC [24–26]. More reports suggest that numerous miRNAs and lncRNAs regulate SOR-R [27, 28]. Similarly, some circRNAs also modulate the SOR-R of HCC. Hence, scientists demonstrated marked alterations in circRNA expressions in a myriad of drug-resistant versus drug-sensitive HCC cells. Collectively, these findings indicate that circRNAs may be used to predict drug efficiency and enhance personalized HCC intervention [29].

Recently, high-throughput sequencing technology has massively augmented the investigation of circRNA expression and mechanism. Employing a GEO microarray assay, we assessed the aberrant expression of circRNAs in SOR-R (SR-HepG2) versus parent-HepG2 cells. Based on our analysis, hsa_circ_0006988 was intricately linked to SOR-R within HCC. To further verify the importance of hsa_circ_0006988 in modulating SOR-R, we conducted a loss-of-function examination by knocking down hsa_circ_0006988 in two SOR-R cell lines (SR-HepG2 and SR-Huh7). Based on our results, hsa_circ_0006988 knockdown dramatically diminished the IC50 of SOR in HCC cells.

CircRNA is known to serve as a ceRNA, which negatively regulates miRNA activity by disrupting its interaction with target mRNA [30, 31]. Herein, we screened hsa_circ_0006988 as a novel miR-15a-5p-interacting circRNA and validated that IGF1 was targeted by miR-15a-5p. Moreover, a literature review revealed that IGF1 is strongly associated with tumorigenesis. Hence, we speculated that IGF1 may serve as a downstream target of hsa_circ_0006988. To identify downstream miRNA targets of hsa_circ_0006988, we employed miR, DLR, and RIP analyses. We demonstrated that hsa_circ_0006988 strongly enhanced SOR-R, primarily via association with miR-15a-5p. We also verified that miR-15a-5p levels were strongly diminished in SOR-R cells relative to SOR-S cells, demonstrating the opposite result of hsa_circ_0006988 overexpression. Subsequently, we validated that hsa_circ_0006988 sequestered miR-15a-5p in HCC cells. First, using bioinformatics-based estimation and a DLR assay, we demonstrated that hsa_circ_0006988 and the IGF1 3 UTR share the same miR-15a-5p response elements, suggesting that they may competitively associated with miR-15a-5p. Second, hsa_circ_0006988 strongly interacted with miR-15a-5p in an AGO2-dependent manner. Third, miR-I reversed the si-hsa_circ_0006988-mediated SOR-S effects. Lastly, hsa_circ_0006988 modulated IGF1 expression via miR-15a-5p modulation. Of note, computational algorithms were used to determine miRNAs, and these results need further investigation to validate them.

## 6. Conclusion

In summary, based on our investigation, hsa_circ_0006988 was a novel chief modulator of the miR-15a-5p/IGF1 axis, and it induced SOR-R in HCC cells. Hsa_circ_0006988 competed with the 3′ UTR of IGF1 for interaction with miR-15a-5p, promoting SOR-R in HCC cells. Our demonstration showed that the hsa_circ_0006988/miR-15a-5p/IGF1 axis modulated SOR-R. This may facilitate the development of novel therapeutic approaches to overcoming SOR-R in HCC.

## Data Availability

The data used to support the findings of this study are available from the corresponding author upon request.
